# Cone Beam Computed Tomography Findings in Calcifying Cystic Odontogenic Tumor Associated with Odontome: A Case Report

**Published:** 2015-12

**Authors:** Tushar Phulambrikar, Sanchita Vilas Kant, Manasi Kode, Shaliputra Magar

**Affiliations:** a Dept. of Oral Medicine & Radiology, Sri. Aurobindo Dental College & PG Institute, India.; b Dept. of Oral Medicine & Radiology, Sri Aurobindo College of Dentistry & PG Institute, Iraq.; c Dept. of Oral Medicine & Radiology, Sri Aurobindo College of Dentistry & PG Institute, India.

**Keywords:** Cone Beam Computed Tomography, Conventional Radiographs, Calcifying Cystic Odontogenic Tumor, Compound Odontome

## Abstract

The calcifying cystic odontogenic tumor (CCOT) is a rare cystic odontogenic neoplasm frequently found in association with odontome. This report documents a case of CCOT associated with an odontome arising in the anterior maxilla in a 28-year-old man. Conventional radiographs showed internal calcification within the lesion but were unable to visualize its relation with the adjacent structures and its accurate extent. In this case cone beam computed tomography (CBCT) could accurately reveal the extent and the internal structure of the lesion which aided the presumptive diagnosis of the lesion as CCOT. This advanced imaging technique proved to be extremely useful in the radiographic assessment and management of this neoplasm of the maxilla.

## Introduction

The calcifying odontogenic cyst (COC) is a rare odontogenic lesion of the jaw, characterized by presence of multiple giant cells and internal calcifications. It was first described by Rywkind in 1932; however, it was in 1962 when Gorlin et al. separately identified it as calcifying odontogenic tumor.[1] COC exhibits both cystic and solid variants. Confusion exists regarding the cyst origin; its variable histology and clinical behavior make it difficult to decide whether the lesion is developmental or reactive.[2]

In the latest description of odontogenic tumors by the World Health Organization (WHO) in 2005, the name of COC was changed to calcifying cystic odontogenic tumor (CCOT), owing to the neoplastic nature of the lesion.[3] The occurrence of CCOT has been described in association with other odontogenic tumors such as ameloblastoma, ameloblastic fibro-odontome, ameloblastic fibroma, calcifying epithelial odontogenic tumor, odontogenic keratocyst, adenomatoid odontogenic tumor, as well as compound and complex odontome. The most common occurrence has been reported in association with an odontome in approximately 24% of cases.[4]

Before performing any surgery, the extent of maxillofacial lesions and their relationship with the surrounding anatomical structures should be carefully examined in order to determine the appropriate treatment plan and to avoid surgical complications. Neither intraoral nor panoramic radiographs provide the three-dimensional (3D) information of the imaged area needed for optimal preoperative planning. In recent years, cone beam computed tomography (CBCT) has emerged as a reliable tool for preoperative radiological assessment of odontogenic cysts and tumors.[5]

The limited number of reported cases indicates the rarity of involvement of maxillary sinus in CCOT.[6]We herein describe a case report of a patient with CCOT associated with an odontome involving the maxillary sinus, focusing on the CBCT findings of the lesions.

## Case Report

A 28-year-old male referred to the Department of Oral Medicine and Radiology, Sri Aurobindo College of Dentistry, Indore, India with a complaint of an asymptomatic swelling and heaviness in the left midface region present for one month. On extraoral evaluation, an asymmetry was observed with an approximately 4×5 cm swelling, involving middle half of left side of the face with obliteration of nasolabial fold. Borders of swelling were indistinct. The overlying skin appeared normal.

**Figure 1:**
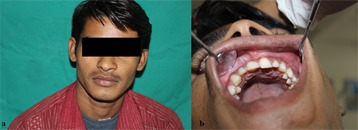
a: Clinical aspect - growth in alveolar process causing buccal cortical expansion with tooth #22 to #26  b: Intraoral picture of the patient showing vestibular obliteration in the second quadrant

Intraoral examination revealed buccal cortical expansion extending from tooth 22 to 26 and palatal expansion with 22 and 23 (Figure 1).

The overlying mucosa appeared normal. On palpation, the swelling was non tender; soft and fluctuant in consistency with 22 and 23 and firm with 24, 25, and 26. No Mobility of teeth was observed. Electric pulp test revealed non vital 23, 24, 25 and delayed pulpal response with 21 and 22.

Intraoral clinical inspection did not reveal any specific finding but signs of moderate gingivitis. The patient did not give incidence of local trauma; medical history was largely noncontributory.

**Figure 2:**
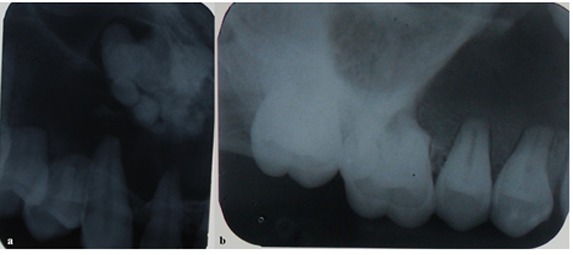
a: Intraoral radiograph showing radiopacity apical to tooth #22, #23  b: Intraoral radiograph showing root resorption with tooth #24, #25, mesiobuccal root of #26

Intraoral periapical radiograph showed a well-defined radiolucency with internal radiopacity extending from periapical region of teeth 11 up to 26 and into the maxillary sinus. There was external root resorption in teeth 24, 25, and 26 (Figures 2).

**Figure 3:**
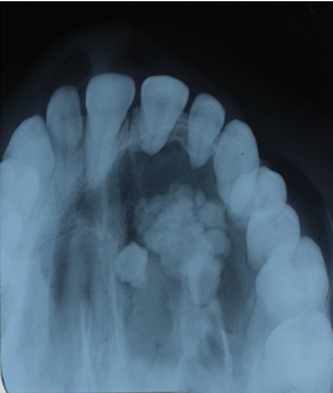
Occlusal radiograph showing radiopacities in left maxillary quadrant

Occlusal radiograph demonstrated unilocular radiolucency extending anterior-posteriorly from mesial of tooth 11 to distal of 26 with multiple ill-defined radiopaque foci of varying density (Figure 3).

**Figure 4:**
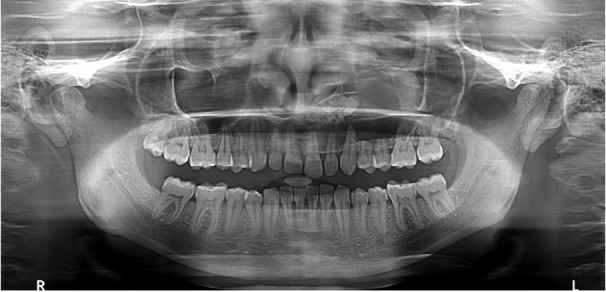
Panoramic image showing mixed radiolucent and opaque lesion in maxillary second quadrant involving maxillary sinus

**Figure 5:**
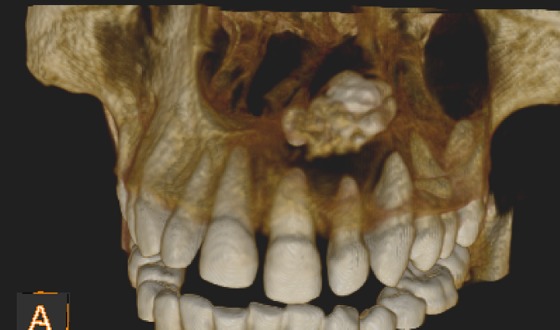
Tridimensional representation of the lesion demonstrating radiopaque foci in relation with tooth #21, 22, 23.

Panoramic radiograph was advised (Figure 4) which demonstrated unicystic cavity extending into the left maxillary sinus, with the area of radiopacity within. CBCT scan was performed to determine the extent of lesion with Kodak CS-9300 using OnDemand software that provided a 10×5 cm field of view, with 360° rotation 90 microns voxel size. The exposure factors were 84 kVp, 20-second rotation time, and 5 mA. CBCT images revealed a large, well-defined unilocular radiolucent expansile lesion with thinned cortical outline involving the entire left side of maxilla, circumscribing the entire quadrant and reaching up to the left infraorbital ridge. Within the lesion, multiple tooth-like high-density structures were observed which depicted radiographic impression of an odontome (Figure 5).

**Figure 6:**
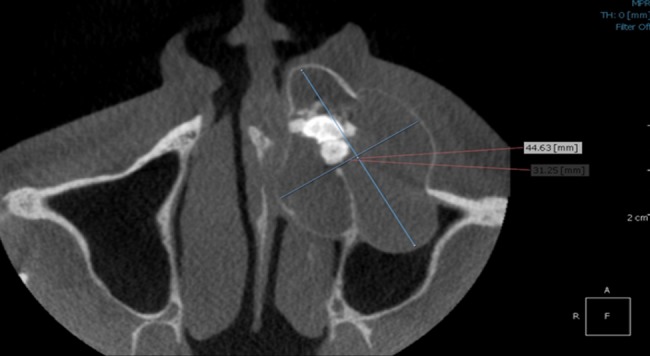
Axial section demonstrated lesion extending from mesial surface of #11 up to mesial surface of #27 with loss of buccal cortical plate in relation with #23, #24, #25 and expansion of the lingual cortical plate.

**Figure 7:**
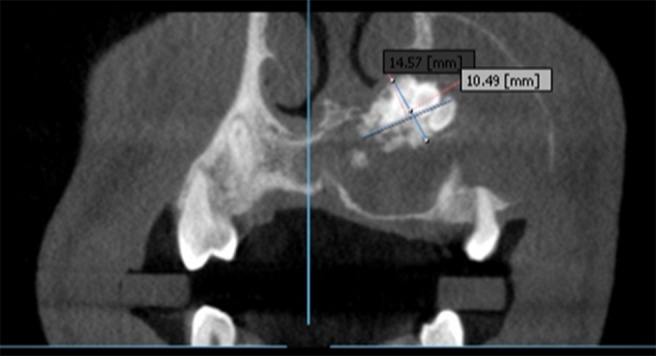
Coronal section of the lesion revealing central opacity measuring 14.57x10.49mm.

Axial section demonstrated lesion extending from the mesial surface of 11 up to the mesial surface of 27 (Figure 6), measuring approximately 44.63 mm anterior- posteriorly and 31.25 mm mediolaterally. Loss of buccal cortical plate was seen in relation with 23, 24, 25 and expansion of lingual cortical plate extending from 11 up to 26. In coronal section the lesion was measured to be mediolaterally 26.63 mm and superior-inferiorly from 33.16 mm. Size of the central opacity was 14.57×10.49 mm (Figure 7).

**Figure 8:**
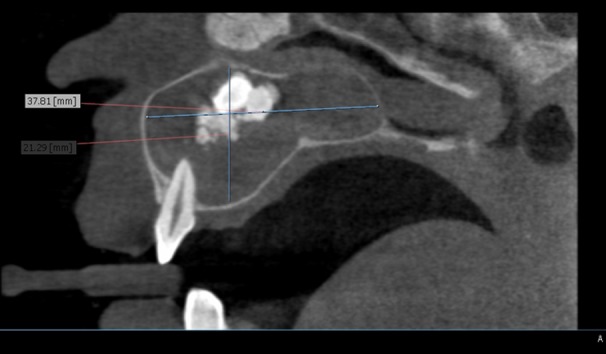
Sagittal section showing the extent of lesion within maxillary sinus

Sagittal section revealed extension of the lesion within the maxillary sinus (Figure 8) with the largest dimension of 37.81 mm anterior-posteriorly up to 21.29 mm superior-inferiorly. The opacity within the lesion was present in relation with 21, 22, 23, and 24 approximately 6.82 mm superiorly from tooth 2. Radiological differential diagnosis included intraosseous calcifying cystic odontogenic tumor, adenomatoid odontogenic tumor, cystic odontome, ossifying fibroma, ameloblastic fibro-odontome, and calcifying epithelial odontogenic tumor.

**Figure 9:**
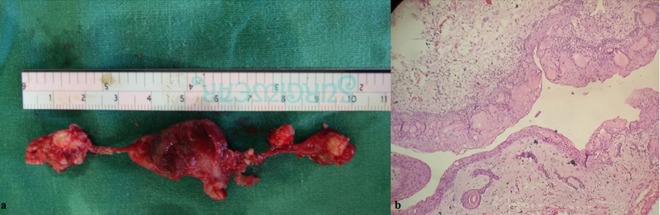
a: Gross specimen  b: Histopathological findings of the cystic wall and fibrous capsule with ameloblastomatous epithelium containing numerous ghost cells.

Surgical enucleation under local anesthesia was performed and a thick cystic wall and mineralized material, compatible with an odontoma in anterior part of the cyst in relation with tooth 21, 22, and 23 was removed, leaving an oval bony defect. (Figure 9a) The H&E staining demonstrated cystic lumen lined by epithelium and connective tissue. Multiple ghost cells were observed. Epithelium was non-keratinized stratified squamous showing basal layer of tall columnar cells with palisaded nucleus suggestive of ameloblast-like cells and overlying stellate reticulum-like cells. The connective tissue showed numerous spindle shaped cells with dense collagen stroma along with Liesegang ring (Figure 9b). Odontogenic rests were also seen within the connective tissue. The histopathological features were suggestive of calcifying cystic odontogenic tumor.

## Discussion

The CCOT represents approximately 2% of all odontogenic tumors,[7] which most frequently occur in the incisor and canine region, with approximately equal frequency of 1:1 within* the *maxilla and mandible.[8] According to Buchner, there was predisposition for the maxilla in Asians; whereas, there was 62% predilection for mandible in whites.[9] CEOT demonstrates bimodal age of distribution with peak incidence in the second and sixth decades of life with equal sex predilection.[8] CCOT is usually an incidental radiographic finding. Radiographically, the lesion appears as a unilocular or multilocular well-defined radiolucency that may contain small irregular calcified bodies of varying sizes associated with an odontome or an unerupted tooth. Root resorption of the associated teeth has been observed in about 75-77 % of the cases.[8]

Recently, various reports have been published about the computed tomography (CT) examinations of these lesions emphasizing its benefits over the plain film radiography to demonstrate the cystic lining of the tumor, along with the internal calcifications. However, very limited data is available in the literature about the value of CBCT in CCOT diagnosis. CBCT scanners have an edge over CT Scans since it uses a low-energy fixed anode, as used for dental panoramic machines and it rotates only once around the patient, using a cone-shaped x-ray beam to capture the data. These changes allow a less expensive, smaller machine that exposes the patient to approximately 20% of the radiation of a helical CT, equal to the exposure received from a set of full-mouth periapical. Also, due to the difference in voxel size, CBCT of a limited area is also very effective in achieving high spatial resolution in comparison with conventional CT.[10]

CCOT are generally unilocular lesions; although 5–13% of cases have shown multilocular appearance. Variable numbers of radio-opaque bodies are seen in about 50% of CCOT cases. It may have regular outline with well-demarcated margins. Early tumors may appear completely radiolucent. With maturation, they develop calcifications which may show mixed radiolucent-radiopaque appearance. Marx et al. in 2003 discussed three patterns of radiopacity with this tumor; first, salt and pepper pattern of flecks, second, fluffy cloudlike pattern throughout, and third, a crescent-shaped pattern on one side of the radiolucency. Our case had well-defined radiopacity within the center of cystic lumen with lobulated surface, regular margins, and well-defined borders not previously discussed in literature. The presence of internal calcification depicted on CBCT images is an important radiographic characteristic for the presumptive diagnosis of the CCOT. The CBCT examination in this case was useful for complete evaluation of the lesions and related structures involving the maxillofacial complex. Visualization of the overlapping adjacent structures represents the CBCT as a challenge in visualization. Another advantage of CBCT examination in the case was the ability to display the extent and complex relationships of the CCOT and the odontome with the associated teeth in 3D, which could not be provided by conventional radiography. In addition, it could be shown by this case report that the CBCT images can offer higher spatial resolution than the conventional X-rays, reducing the radiation dose and also guiding the treatment of this lesion. In summary, we reported a rare case of CCOT associated with an odontoma. Internal calcification detected on CBCT images aided the presumptive diagnosis of the lesion as CCOT, which was subsequently diagnosed histopathologically as CCOT. CBCT was also useful in determining the extent and relationship of the CCOT and odontome with the adjacent structures. 
